# Disaster preparedness knowledge, attitude, and practice among Emergency Department staff in Ethiopian Hospitals

**DOI:** 10.1016/j.afjem.2026.100967

**Published:** 2026-03-27

**Authors:** Yonnas Nakachew Baleh, Mikiyas G. Teferi, Hywet Engida, Kebron W. Aweke, Demmelash Gezahegn Nigatu

**Affiliations:** a Department of Emergency Medicine and Critical Care, School of Medicine, College of Health Sciences, Addis Ababa University, Addis Ababa, Ethiopia; b School of Medicine, College of Health Sciences, Addis Ababa University, Addis Ababa, Ethiopia

**Keywords:** Disaster preparedness education, Emergency department, Curriculum development, Health education, Knowledge assessment, Ethiopia

## Abstract

**Introduction:**

Disasters cause significant morbidity and mortality across Ethiopia, yet Emergency Department health professionals often lack adequate disaster-preparedness training. This study assessed knowledge, attitude, and practice of disaster preparedness among health professionals in the Emergency Departments of three tertiary hospitals in Addis Ababa, Ethiopia, to inform emergency medicine education and training.

**Methods:**

A descriptive cross-sectional study was conducted from March to August 2021 among 197 health professionals. Data were collected using a structured questionnaire adapted from previously published disaster-preparedness Knowledge–Attitude–Practice instruments. Knowledge, attitude, and practice were assessed using validated scoring systems. Bivariate and multivariable logistic regression identified independent predictors.

**Results:**

Among 197 participants (of which 72.6% were nurses), with mean age 29.3 years, 53.3% demonstrated inadequate knowledge, 91.9% positive attitudes, and 59.4% inadequate practice. Only 29.4% had received disaster training, 48.7% were unaware of hospital disaster plans, and 31.5% had participated in drills within the past year. Prior disaster training independently predicted adequate knowledge (AOR 3.210, 95% CI 1.324–7.782) and adequate practice (AOR 6.281, 95% CI 2.442–16.154).

**Conclusion:**

Despite positive attitudes, disaster-preparedness knowledge and practical readiness among Ethiopian Emergency Department professionals remain suboptimal. The strong association between training and preparedness highlights the need for structured disaster-preparedness education and regular simulation-based training to strengthen response capacity.

## African relevance

• Identifies disaster-preparedness training gaps among emergency-department professionals in an African setting.
• Provides context-specific data to inform disaster-preparedness education in resource-limited emergency-care systems.
• Highlights the association between prior training and higher preparedness among ED staff.
• Contributes baseline evidence for strengthening disaster-preparedness capacity in sub-Saharan Africa.
• Supports consideration of disaster-preparedness training within emergency-care education frameworks.

## Introduction

Disasters, whether natural or human-made, continue to challenge health systems worldwide, causing substantial loss of life, social disruption, and economic damage. Over the past two decades, their frequency and severity have increased, disproportionately affecting low- and middle-income countries with fragile health infrastructure and limited emergency response capacity [[1], [2], [3], [4], [5]]. In sub-Saharan Africa, recurrent droughts, floods, epidemics, and mass-casualty incidents have repeatedly strained health systems, underscoring the need for resilient and well-prepared emergency care services [6,7].

Hospitals and Emergency Departments (EDs) play a central role in disaster response and must maintain functional readiness and adequately trained personnel to manage sudden surges in patient demand [8,9]. ED professionals are frontline responders during disasters, and their effectiveness depends largely on their knowledge, attitude, and practice of disaster management [10].

Evidence from multiple settings has shown that healthcare workers often demonstrate limited disaster-preparedness knowledge and experience despite generally positive attitudes, while structured training and simulation-based education improve preparedness [[11], [12], [13], [14], [15]]. In Ethiopia, increasing disaster risks and the evolving emergency-medicine system highlight the importance of institutional readiness; however, existing studies indicate gaps in disaster planning, training, and awareness of institutional protocols [[16], [17], [18]].

Although previous Ethiopian studies have examined disaster preparedness among general hospital staff, evidence specific to ED professionals remains limited [19]. Because EDs function as primary frontline response units during disasters and mass-casualty incidents, assessing preparedness within this group is essential. This study therefore aimed to assess the knowledge, attitude, and practice of disaster preparedness among ED health professionals across tertiary hospitals in Addis Ababa, Ethiopia, and to identify factors associated with preparedness to inform training and capacity-building in emergency care.

## Methods

### Study design and setting

A descriptive cross-sectional study was conducted from March to August 2021 to assess disaster-preparedness knowledge, attitude, and practice among ED professionals. It was carried out in three hospitals in Addis Ababa, Ethiopia: Tikur Anbessa Specialized Hospital (TASH), St Paul’s Hospital Millennium Medical College (SPHMMC), and Addis Ababa Burn Emergency and Trauma Hospital (AaBET). TASH and SPHMMC are tertiary referral centers and serve as the country’s primary training sites for emergency medicine. AaBET Hospital is a specialized emergency and trauma center affiliated with SPHMMC, providing dedicated care for burn, trauma, and critical emergency conditions.

### Study population and sample

The study population included all nurses, emergency-medicine residents, and physicians working in the adult EDs of the three hospitals during the study period. Staff members on leave, working less than one year in the ED, or temporarily assigned were excluded. A census sampling approach was employed to include all 320 eligible professionals.

### Data collection instrument

Data were collected using a structured, self-administered questionnaire adapted from validated Knowledge–Attitude–Practice instruments on disaster preparedness. The tool included four sections: socio-demographic and professional characteristics; 13 knowledge items on disaster concepts, preparedness, response, and institutional plans; 17 attitude statements measured on a five-point Likert scale; and 10 practice items assessing participation in training, drills, and response activities. The questionnaire was prepared in English and pretested on 10% of the sample at a different tertiary hospital to ensure clarity and internal consistency, with revisions made accordingly. Two trained data collectors distributed and collected sealed questionnaires during duty hours under supervision of the principal investigator. The knowledge component focused on hospital-based disaster preparedness, including institutional plans, emergency-department response to mass-casualty incidents, and core disaster-response principles; first-aid items referred to immediate disaster response and did not assess out-of-hospital care.

### Measurement and scoring

Knowledge was scored as one point for each correct response and zero for incorrect or “don’t know” answers; scores at or above the mean were classified as adequate knowledge. Attitude was measured using a five-point Likert scale, with scores above the mean indicating a positive attitude. Practice was assessed similarly, with scores at or above the mean considered adequate practice. Independent variables included socio-demographic factors (age, sex, profession, years of experience, educational level, and prior disaster training) and institutional factors (hospital, workload, and resource availability), while dependent variables were knowledge, attitude, and practice scores. In the absence of a universal competency threshold, knowledge classification was based on the mean score of the validated instrument, consistent with prior Knowledge, Attitude, and Practice studies.

### Data management and analysis

Data were checked for completeness, coded, and entered into Epi Info version 7 and exported to SPSS version 26 for analysis. Descriptive statistics were used to summarize categorical and continuous variables, presented as frequencies, percentages, means, and standard deviations. Associations between independent variables and outcomes (knowledge, attitude, and practice) were first assessed using bivariate logistic regression. Variables with a p-value < 0.25 in bivariate analysis were entered into multivariate logistic regression to identify independent predictors. Odds ratios with 95% confidence intervals were calculated, and statistical significance was set at *p* < 0.05.

### Quality assurance

Completed questionnaires were returned in sealed envelopes and reviewed for completeness after collection; the investigator had no access to responses during data collection. Data entry was verified through double-entry checks Questionnaires with substantial missing data (>10% incomplete items) were excluded based on predefined criteria, and questionnaires were not re-administered to avoid potential response bias. Internal consistency was confirmed using Cronbach’s alpha (α = 0.82).

### Ethical considerations

Ethical approval was obtained from the Institutional Review Boards of TASH (AAU/ECCM-033/2021) and SPHMMC (SPHMMC/EM/114/21). Written informed consent was obtained from all participants. No personal identifiers were collected, and data were kept confidential. All participating institutions consented to being identified in this publication.

## Results

Of 320 eligible ED professionals, 230 returned questionnaires (response rate 71.9%), and 197 complete responses were included in the final analysis (completion rate 61.6%). Participants had a mean age of 29.3 years (SD 3.9), and 58.8% were male. Nurses constituted the majority of respondents, while physicians and residents accounted for the remainder. Most respondents reported no prior disaster-management training and had less than five years of ED experience (Table 1).

**Table 1:** Socio-demographic characteristics of participants (*n* = 197).

*Socio-demographic variables*		*Male*	*Female*	*Total (n/%)*	*Mean ± S D*
**Age category**	20 – 24	1	9	10 (5.1)	29.34±3.895
	25 – 29	65	34	99 (50.3)	
	30 – 34	36	27	63 (32)	
	35 – 39	14	9	23 (11.7)	
	≥ 40	0	2	2 (1.0)	

**Working hospital**	TASH	41	28	69 (35.0)	
	SPHMMC	51	24	75 (38.1)	
	AaBET Hospital	24	29	53 (26.9)	

**Level of education**	Specialty certificate	8	7	15 (7.6)	
	Third year resident	12	2	14 (7.1)	
	Second year resident	22	3	25 (12.7)	
	Master	6	10	16 (8.1)	
	Bachelor’s degree (university level)	65	55	120 (60.9)	
	Diploma	3	4	7 (3.6)	

**ED work experience**	<2 years	38	19	57 (28.9)	3.39 ± 1.140
	2 to <5 years	60	39	99 (50.3)	
	5 to 10 years	16	22	38 (19.3)	
	>10years	2	1	3 (1.5)	

**Disaster training**	Yes	37	21	58 (29.4)	
	No	79	60	139 (70.6)	

**Total**		116	81	197(100)	

ED = Emergency Department; TASH = Tikur Anbessa Specialized Hospital; SPHMMC = St Paul’s Hospital Millennium Medical College; AaBET = Addis Ababa Burn Emergency and Trauma Hospital; SD = standard deviation.

### Knowledge of disaster preparedness

Overall, fewer than half of respondents demonstrated adequate knowledge of disaster preparedness. While most participants correctly identified the definition of a disaster and the importance of immediate first aid during disasters, awareness of institutional preparedness procedures was limited. Only about half of respondents were aware that their hospital had a disaster plan, and fewer knew where the plan was located or the designated evacuation area. Slightly more than half had ever seen or participated in a disaster drill in the ED (Table 2).

**Table 2:** Knowledge of Emergency Department professionals regarding disaster preparedness and planning.

	Yes/correct answer (*n* = 197)	Percentage
1. Correctly identified the meaning of a disaster	154	78.2
2. Correctly identified the meaning of disaster preparedness	132	67.0
3. First aid should be given immediately during disaster	181	91.9
4. Bystanders or community members can provide first aid during disasters	143	72.6
5. The hospital has a disaster plan	101	51.3
6. Knew where to find a copy of the plan in the department	52	26.4
7. Know what a hospital disaster plan should contain	55	27.9
8. Know when an alert status for an emergency management plan is activated	107	54.3
9. Know the specific place for evacuation for patients during disaster event	83	42.1
10. Familiar with disaster drills or simulations	110	55.8
11. Have seen emergency/disaster drill occurring in the ED	109	55.3

In regression analysis, several factors were associated with adequate knowledge (**Supplementary Table S1**). Male participants were more likely to demonstrate adequate knowledge than females. Working hospital was also associated with knowledge, with participants from SPHMMC and AaBET showing higher odds of adequate knowledge compared with those from TASH. Prior disaster-management training and adequate disaster-preparedness practice were significant predictors of knowledge after adjustment, whereas educational level was not independently associated with knowledge.

### Attitude toward disaster preparedness

Overall, most respondents demonstrated a positive attitude toward disaster preparedness (91.9%). Participants broadly supported hospital disaster readiness and the need for regular training and simulation exercises, and most agreed that disaster-management training should be integrated into medical and nursing curricula. However, fewer respondents believed that a disaster was likely to occur in their own hospital. Attitude scores did not differ significantly across gender, profession, or hospital (Table 3).

**Table 3:** Attitudes of participants toward disaster preparedness.

Item	Attitude statement	Very much disagree n (%)	Disagree n (%)	Neutral n (%)	Agree n (%)	Very much agree n (%)
1	The EDs should be adequately prepared should a disaster occur	15 (7.6)	18 (9.1)	17 (8.6)	71 (36.0)	76 (38.6)
2	Drills should be conducted in the EDs	12 (6.1)	17 (8.6)	14 (7.1)	94 (47.7)	60 (30.5)
3	Training is necessary for all health professionals	12 (6.1)	9 (4.6)	9 (4.6)	59 (29.9)	108 (54.8)
4	Disaster simulations should occur frequently in the ED	4 (2.0)	22 (11.2)	25 (12.7)	92 (46.7)	54 (27.4)
5	The hospital should have disaster plans	8 (4.1)	11 (5.6)	15 (7.6)	74 (37.6)	89 (45.2)
6	The hospital should assess the importance of vulnerability	5 (2.5)	10 (5.1)	21 (10.7)	87 (44.2)	74 (37.6)
7	The hospital is unlikely to be affected by disasters	68 (34.5)	55 (27.9)	25 (12.7)	42 (21.3)	7 (3.6)
8	Disaster planning is only for the hospital’s administration	72 (36.5)	66 (33.5)	19 (9.6)	29 (14.7)	11 (5.6)
9	Disaster management is for nurses and doctors only	77 (39.1)	81 (41.1)	7 (3.6)	23 (11.7)	9 (4.6)
10	Disasters are unlikely to happen in our hospital	70 (35.5)	53 (26.9)	21 (10.7)	45 (22.8)	8 (4.1)
11	I need to know about disasters and disaster plans	5 (2.5)	5 (2.5)	17 (8.6)	69 (35.0)	101 (51.3)
12	Disaster training should be part of education in Addis Ababa teaching hospitals	6 (3.0)	7 (3.6)	29 (14.7)	69 (35.0)	86 (43.7)

### Practice of disaster preparedness

Overall, 40.6% of participants demonstrated adequate disaster-preparedness practice. Participation in disaster drills and ongoing disaster-management training was relatively low, and only a small proportion reported that hospital disaster plans were regularly updated. However, a majority of respondents (61.4%) reported having managed at least one disaster or mass-casualty incident during their professional career (Table 4).

**Table 4:** Practices of participants toward disaster preparedness and hospital disaster plans.

Item	Practice statement	Yes / Practiced n (%)
1	Practiced or drilled on what to do in an emergency or disaster situation in the past 1 year	62 (31.5)
2	Participated in ongoing disaster management training in the hospital	36 (18.3)
3	Seen or heard the disaster plan being periodically updated by the hospital authority	21 (10.7)
4	Faced or responded to a real disaster in the ED during employment	121 (61.4)
5	Member of the hospital disaster management team	74 (37.6)
6	Taken first aid training, such as basic life support, within the past one year	127 (64.5)
7	Believes they have sufficient practical skills to manage emergencies or disasters	90 (45.7)

**Fig. 1:**
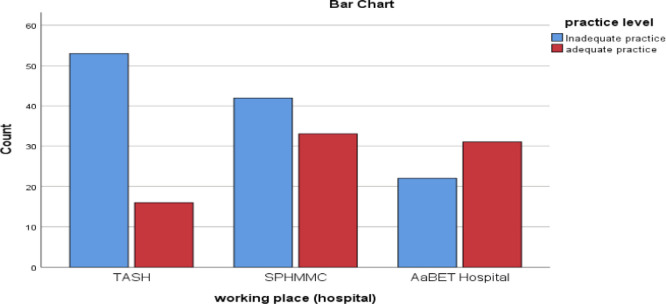
Distribution of adequate and inadequate disaster-preparedness practice among ED professionals across the three participating hospitals. **TASH** = Tikur Anbessa Specialized Hospital; **SPHMMC** = St Paul’s Hospital Millennium Medical College; **AaBET** = Addis Ababa Burn Emergency and Trauma Hospital; **ED** = Emergency Department.

Membership in hospital disaster committees was low, with only 37.6% identifying themselves as members. About 46% of participants believed they were personally competent to respond effectively in a disaster situation. Staff from AaBET and SPHMMC reported better practice scores than those from TASH (Fig. 1).

### Factors associated with practice

Multivariable logistic regression identified several factors independently associated with adequate disaster-preparedness practice (Table 5). Working hospital was significantly associated with practice, with participants from SPHMMC (AOR 5.10, 95% CI 1.71–15.23) and AaBET (AOR 8.46, 95% CI 2.81–25.52) more likely to report adequate practice compared with those from TASH. Prior disaster-management training was a strong predictor, with trained participants over six times more likely to demonstrate adequate practice than those without training (AOR 6.28, 95% CI 2.44–16.15). Adequate knowledge was also independently associated with practice (AOR 3.35, 95% CI 1.52–7.39). In contrast, profession and years of ED experience were not significantly associated with practice after adjustment.

**Table 5:** Multivariable logistic regression analysis showing predictors of adequate disaster-preparedness practice among ED professionals (*n* = 197).

Variable	Adequate practice n (%)	AOR (95% CI)	p-value
**Working hospital**			
TASH	16 (23.2)	1 (reference)	—
SPHMMC	33 (44.0)	5.10 (1.71–15.23)	<0.001
AaBET	31 (58.5)	8.46 (2.81–25.52)	<0.001
**Disaster-management training**			
No	36 (25.9)	1 (reference)	—
Yes	44 (75.9)	6.28 (2.44–16.15)	<0.001
**Knowledge level**			
Inadequate	22 (21.0)	1 (reference)	—
Adequate	58 (63.0)	3.35 (1.52–7.39)	0.003
**Profession**			
Staff nurse	55 (38.5)	1 (reference)	—
Second-year resident	7 (28.0)	0.83 (0.07–9.62)	0.168
Third-year resident	9 (64.3)	9.59 (0.76–121.22)	0.168
Emergency physician	9 (60.0)	1.91 (0.16–22.22)	0.168
**ED experience**			
<2 years	13 (22.8)	1 (reference)	—
2 to <5 years	45 (45.5)	2.92 (0.85–10.02)	0.333
5–10 years	21 (55.3)	3.80 (0.97–14.83)	0.333
>10 years	1 (33.3)	0.89 (0.02–35.03)	0.333

**Abbreviations**: AOR = adjusted odds ratio; CI = confidence interval; ED = emergency department; TASH = Tikur Anbessa Specialized Hospital; SPHMMC = St Paul’s Hospital Millennium Medical College; AaBET = Addis Ababa Burn Emergency and Trauma Hospital.

## Discussion

This study assessed disaster-preparedness knowledge, attitude, and practice among ED professionals in three tertiary hospitals in Ethiopia. While most participants demonstrated positive attitudes toward disaster preparedness, both knowledge and practical readiness were limited. Prior disaster-management training and institutional context were key predictors of preparedness, and adequate knowledge was associated with better practice. These findings highlight important educational and system-level gaps in disaster preparedness among frontline emergency-care providers.

The observed gaps in disaster-preparedness knowledge and practice, despite generally positive attitudes, suggest that awareness alone does not translate into operational readiness. Prior disaster-management training emerged as the strongest predictor of both knowledge and practice, underscoring the critical role of structured training, simulation exercises, and regular drills in building practical preparedness among emergency-department professionals. The association between knowledge and practice further indicates that improving educational exposure may enhance real-world response capability. Differences observed across hospitals may reflect variability in training opportunities, institutional preparedness, and exposure to disaster response, highlighting the influence of system-level factors on preparedness. Collectively, these findings suggest that strengthening structured training and institutional preparedness mechanisms is essential to improving disaster-response capacity among frontline emergency-care providers.

The low level of disaster-preparedness knowledge observed in this study is consistent with reports from Bangladesh, Rwanda, and Malaysia, where healthcare workers demonstrated limited preparedness despite positive attitudes toward training [[11], [12], [13],^20^,^21^]. In contrast, higher preparedness in Saudi Arabia and Japan has been linked to mandatory drills and structured continuing education [14,22]. Similar gaps across Africa, including Nigeria, South Africa, and Ethiopia, highlight persistent deficiencies in training, protocol awareness, and institutional coordination, suggesting that disaster-preparedness systems remain underdeveloped in many sub-Saharan African settings [6,9,17,18].

The gaps in knowledge and practice observed in this study may reflect the limited integration of disaster-management content into medical and nursing curricula and the relatively young status of emergency medicine as a specialty in Ethiopia. Few hospitals conduct regular drills or refresher courses, and disaster committees often lack resources to sustain training initiatives. Introducing periodic simulation exercises and multidisciplinary workshops can improve teamwork, confidence, and coordination during emergencies. Embedding structured disaster-preparedness modules into undergraduate and postgraduate programs would align Ethiopia with international frameworks advocating resilient health systems.

From a policy perspective, hospitals may ensure that disaster-management plans are regularly updated, accessible to staff, and supported by leadership. The Ministry of Health could establish national standards for disaster training and integrate preparedness indicators into accreditation and quality-monitoring systems. Strengthening interdepartmental communication, establishing rapid-response teams, and allocating specific budgets for training and drills may improve institutional resilience and response capability. These measures are cost-effective ways to enhance national readiness for future emergencies.

This study has several limitations. First, its cross-sectional design limits causal inference and does not allow assessment of changes over time. Second, reliance on self-reported questionnaires may have introduced recall or social-desirability bias. Third, although data were collected in 2021, no major systemic reforms in disaster-preparedness training or institutional frameworks have occurred in the participating hospitals since that time; however, evolving health-system dynamics may still influence current preparedness levels. Fourth, the study was conducted in three tertiary hospitals in Addis Ababa and may not represent smaller regional facilities. Because nurses comprised most respondents, the findings may primarily reflect nursing preparedness and may not generalise equally to physicians, although this distribution mirrors the ED workforce and remains operationally relevant. Despite these limitations, the study provides an important baseline for guiding training initiatives and future research on hospital disaster preparedness.

Future research should evaluate the effectiveness of simulation-based training and examine organizational factors influencing preparedness, including leadership, resource allocation, and interdepartmental coordination. Longitudinal and multicentre studies are needed to determine whether educational and policy interventions lead to sustained improvements in ED readiness and patient outcomes.

## Conclusion

ED professionals in Ethiopian teaching hospitals showed positive attitudes toward disaster preparedness, but knowledge and practical readiness were limited, with low exposure to formal training and institutional drills. These findings highlight the need for structured disaster-preparedness education and regular simulation-based training within hospitals. Strengthening training and institutional preparedness systems is essential to improve emergency-department capacity to respond effectively to disasters and mass-casualty events.

### Dissemination of results

Study findings have been submitted as a thesis to the Department of Emergency Medicine, Addis Ababa University College of Health Sciences, in partial fulfillment of residency program requirements. Results were presented to clinical staff and administration of the participating EDs to inform disaster-preparedness training initiatives. Findings were presented at the African Conference on Emergency Medicine in Botswana in November 2024.

## CRediT authorship contribution statement

**YB:** Conceptualization, Methodology, Investigation, Data curation, Formal analysis, Project administration, Writing – original draft; **MT:** Methodology, Validation, Writing – review & editing; **HE:** Conceptualization, Supervision, Writing – review & editing; **KA:** Methodology, Validation, Writing – review & editing; **DN:** Supervision, Validation, Writing – review & editing. All authors approved the final version to be published and agreed to be accountable for all aspects of the work.

## Availability of data and material

The datasets generated and/or analyzed during the current study are available from the corresponding author upon reasonable request.

## Funding

None received.

## Declaration of competing interest

The authors declare no conflict of interest.

## Appendix Supplementary materials

Image, application 1

## References

[1] Krichen M., Abdalzaher M.S., Elwekeil M., Fouda M.M. (2024). Managing natural disasters: an analysis of technological advancements, opportunities, and challenges. Internet Things Cyberphys Syst.

[2] Amaratunga D., Anzellini V., Guadagno L., Hagen J.S., Komac B., Krausmann E. (2023). https://www.undrr.org/rar/rar-2023-europe-and-central-asia.

[3] World Health Organization (2019). https://www.who.int/publications-detail-redirect/health-emergency-and-disaster-risk-management-framework.

[4] Kaji A., Koenig K.L., Bey T. (2006). Surge capacity for healthcare systems: a conceptual framework. Acad Emerg Med.

[5] World Bank (2021). https://www.gfdrr.org/en/feature-story/building-resilient-health-systems-africa.

[6] Usoro A., Mehmood A., Rapaport S., Ezeigwe A.K., Adeyeye A., Akinlade O. (2023). A scoping review of the essential components of emergency medical response systems for mass casualty incidents. Disaster Med Public Health Prep.

[7] Aitsi-Selmi A., Egawa S., Sasaki H., Wannous C., Murray V. (2015). The Sendai framework for disaster risk reduction: renewing the global commitment to people’s resilience, health, and well-being. Int J Disaster Risk Sci.

[8] Schultz C.H., Koenig K.L., Lewis R.J. (2003). Implications of hospital evacuation after the Northridge, California, earthquake. N Engl J Med.

[9] Fekadu S.T., Gebrewahid A.L., Mankoula W., Eteng W., Lokossou V., Kawe Y. (2023). Public health emergency operations centres in Africa: a cross-sectional study assessing the implementation status of core components and areas for improvement. BMJ Open.

[10] Subbarao I., Lyznicki J.M., Hsu E.B., Gebbie K.M., Markenson D., Barzansky B. (2008). A consensus-based educational framework and competency set for disaster medicine and public health preparedness. Disaster Med Public Health Prep.

[11] Rahman M.M., Rahman M.S., Jerin T. (2023). Social vulnerability to earthquake disaster: insights from the 48th ward of Dhaka South City, Bangladesh. Env Hazards.

[12] Alzahrani F., Kyratsis Y. (2017). Emergency nurse disaster preparedness during mass gatherings: a cross-sectional survey in hospitals in Mecca, Saudi Arabia. BMJ Open.

[13] Nasir N.M., Zahari H.M., Husain R. (2023). A systematic literature review on logistics information needs for sharing in Malaysian disaster management. Sustainability.

[14] Kapucu N., Hawkins C.V., Rivera F.I. (2013). Disaster preparedness and resilience for rural communities. Risk Hazards Crisis Public Policy.

[15] Khaddage-Soboh N., Tawil S. (2023). Navigating the crisis: a review of COVID-19 research and the importance of academic publications. Heliyon.

[16] Kebede G.F., Gurmu B.W., Weldegebriel Z.B. (2025). Integrating climate change adaptation, disaster risk reduction, and social protection for enhanced resilience: evidence from the Somali region, Ethiopia. J Env Dev.

[17] Berhanu N., Abrha H., Ejigu Y., Woldemichael K. (2016). Knowledge, experiences and training needs of health professionals about disaster preparedness and response in southwest Ethiopia. Ethiop J Health Sci.

[18] Gudina E.K., Siebeck M., Eshete M.T. (2021). Evidence gaps and challenges in the fight against COVID-19 in Africa: scoping review of the Ethiopian experience. Risk Manag Heal Policy.

[19] Habte A., Addisie A., Azazh A. (2018). Assessment of knowledge, attitude and practice of disaster preparedness among Tikur Anbessa Specialized Hospital health care workers, Addis Ababa, Ethiopia. Am J Nurs Sci.

[20] Zewudie A., Regasa T., Kebede O., Abebe L., Feyissa D., Ejata F. (2021). Healthcare professionals’ willingness and preparedness to work during COVID-19 in southwest Ethiopia. Risk Manag Heal. Policy.

[21] Walles S., Chona E.Z., Ndile M.L., Ramadhani F.B. (2023). Knowledge, attitude and practices of health care providers towards disaster and emergency preparedness in Mtwara, Tanzania. Rwanda J Med Health Sci.

[22] Munasinghe N.L., O’Reilly G., Cameron P. (2022). Establishing the domains of a hospital disaster preparedness evaluation tool: a systematic review. Prehosp Disaster Med.

