# Supplementary material for: Disaster preparedness knowledge, attitude, and practice among Emergency Department staff in Ethiopian Hospitals
Source: Afr J Emerg Med. 2026 Mar 27;16(2):100967. doi: 10.1016/j.afjem.2026.100967 (PMC13054011; doi:10.1016/j.afjem.2026.100967)
Supplement: Supplementary file 1 [file mmc1.docx]

**Supplementary Table S1** Bivariate and multiple logistic regression analysis showing predictors of knowledge levels (n=197)

| **Independent Variable** | **adequate Knowledge n (%)** | **Bivariate COR (95% CI)** | **p-value** | **Multivariate AOR (95% CI)** | **p-value** |
| --- | --- | --- | --- | --- | --- |
| **Gender** |  |  | **.024** |  | **.014** |
| Male | 62 (53.4) | 1.952 (1.093–3.486) |  | 2.777 (1.230–6.270) |  |
| Female | 30 (37.0) | 1 |  | 1 |  |
| **Working hospital** |  |  | **.021** |  | **.035** |
| TASH | 23 (33.3) | 0.5 |  | 1 |  |
| SPHMMC | 39 (52.0) | 2.167 (1.103–4.256) |  | 2.814 (1.085–7.294) |  |
| AaBET | 30 (56.6) | 2.609 (1.246–5.460) |  | 3.634 (1.250–10.566) |  |
| **Level of education** |  |  | **.030** |  | **.152** |
| Specialty certificate | 11 (73.3) | 3.667 (0.557–24.132) |  | 4.595 (0.379–55.698) |  |
| Third year resident | 8 (57.1) | 1.778 (0.284–11.120) |  | 2.445 (0.210–28.401) |  |
| Second year resident | 12 (48.0) | 1.231 (0.227–6.671) |  | 1.531 (0.164–14.294) |  |
| Master | 12 (75.0) | 4.000 (0.612–26.123) |  | 8.364 (0.679–103.012) |  |
| Bachelor’s degree (university level) | 46 (38.3) | 0.829 (0.177–3.872) |  | 1.188 (0.168–8.419) |  |
| Diploma | 3 (42.9) | 1 |  | 1 |  |
| **Disaster training** |  |  | **<0.001** |  | **.010** |
| Yes | 45 (77.6) | 6.776 (3.331–13.784) |  | 3.210 (1.324–7.782) |  |
| No | 47 (33.8) | 1 |  | 1 |  |
| **Practice level** |  |  | **<0.001** |  | **.002** |
| Adequate | 58 (72.5) | 6.436 (3.419–12.115) |  | 3.506 (1.561–7.872) |  |
| Inadequate | 34 (29.1) | 1 |  | 1 |  |

**Abbreviations:** AOR = adjusted odds ratio; COR = crude odds ratio; CI = confidence interval; TASH = Tikur Anbessa Specialized Hospital; SPHMMC = St Paul’s Hospital Millennium Medical College; AaBET = Addis Ababa Burn Emergency and Trauma Hospital.

**QUESTIONNAIRE**

**PART I: Socio-Demographic Information**

1. **Age:** ______ years
2. **Sex**

- ☐ Male
- ☐ Female

1. **Religion**

- ☐ Orthodox
- ☐ Muslim
- ☐ Protestant
- ☐ Other: ___________

1. **Marital Status**

- ☐ Single
- ☐ Married
- ☐ Divorced / Separated / Widowed

1. **Do you have children?**

- ☐ Yes
- ☐ No

1. **Working Place (Hospital)**

- ☐ TASH
- ☐ SPHMMC
- ☐ AaBET Hospital

1. **Professional Category**

- ☐ Staff Nurse
- ☐ 2nd Year Resident
- ☐ 3rd Year Resident
- ☐ Emergency Physician

1. **Level of Education**

- ☐ Diploma
- ☐ Degree
- ☐ Master’s Degree
- ☐ 2nd Year Resident
- ☐ 3rd Year Resident
- ☐ Specialty Certificate

1. **Emergency Department Work Experience**

- ☐ <2 years
- ☐ 2 to <5 years
- ☐ 5–10 years
- ☐ >10 years

1. **Have you received disaster management training before?**

- ☐ Yes
- ☐ No

**PART II:**

**Knowledge Regarding Disaster Preparedness**

**1. What is a disaster? (Select ONE answer)**

A. ☐ An evaluation of the probability of occurrence and magnitude of hazards 
B. ☐ A serious disruption of a community or society causing widespread human, material, economic, or environmental loss exceeding the ability of the community to cope using its own resources 
C. ☐ A possible threat or source of exposure to injury, harm, or loss

**2. What is disaster preparedness? (Select ONE answer)**

A. ☐ Actions taken in anticipation of an emergency to facilitate rapid and effective response 
B. ☐ System of procedures and audits to ensure technical and reporting quality 
C. ☐ A process through which activities are undertaken at appropriate levels

**Knowledge Questions**

1. **Do you know that your hospital has an emergency/disaster plan?**

- ☐ Yes
- ☐ No
- ☐ I don’t know (Skip next question)

3.1 **Do you know where to find a copy of the disaster plan in your department?**

- ☐ Yes
- ☐ No

1. **Do you know what a hospital disaster plan should contain?**

- ☐ Yes
- ☐ No

4.1 **List some contents of the hospital disaster plan**

1. **Do you know when an alert status for an emergency management plan is activated in your hospital?**

- ☐ Yes
- ☐ No

1. **Do you know the specific place for evacuation of patients during a disaster event?**

- ☐ Yes
- ☐ No

1. **Do you know what disaster drills or simulations are?**

- ☐ Yes
- ☐ No

1. **Have you seen any emergency/disaster drill occurring in your emergency department?**

- ☐ Yes
- ☐ No

1. **When should first aid be given during a disaster?**

- ☐ Immediately
- ☐ Only in hospital settings

1. **Who should give first aid during a disaster?**

- ☐ Only healthcare workers
- ☐ Bystanders including the community

1. **How do you rate your current knowledge and skill in managing disaster events?**

- ☐ Very good
- ☐ Good
- ☐ Poor

**PART III: Attitude Toward Disaster Preparedness**

**Instructions:** Indicate your level of agreement.

| **Statement** | **Very much disagree** | **Disagree** | **Neutral** | **Agree** | **Very much agree** |
| --- | --- | --- | --- | --- | --- |
| 1. The emergency department should be adequately prepared to manage disasters | ☐ | ☐ | ☐ | ☐ | ☐ |
| 2. Disaster drills should be conducted in the emergency department | ☐ | ☐ | ☐ | ☐ | ☐ |
| 3. Emergency department health professionals need disaster training | ☐ | ☐ | ☐ | ☐ | ☐ |
| 4. Disaster simulations should occur frequently | ☐ | ☐ | ☐ | ☐ | ☐ |
| 5. Hospitals should have disaster preparedness plans | ☐ | ☐ | ☐ | ☐ | ☐ |
| 6. Hospitals should assess their vulnerability to disasters | ☐ | ☐ | ☐ | ☐ | ☐ |
| 7. The hospital is unlikely to be affected by disasters | ☐ | ☐ | ☐ | ☐ | ☐ |
| 8. Disaster planning is only for hospital administrators | ☐ | ☐ | ☐ | ☐ | ☐ |
| 9. Disaster management is only for doctors and nurses | ☐ | ☐ | ☐ | ☐ | ☐ |
| 10. Disasters are unlikely to happen in our hospital | ☐ | ☐ | ☐ | ☐ | ☐ |
| 11. I need to know about disasters and disaster plans | ☐ | ☐ | ☐ | ☐ | ☐ |
| 12. Disaster training should be part of education in teaching hospitals | ☐ | ☐ | ☐ | ☐ | ☐ |

**Willingness to Report During Infectious Disease Outbreak**

| **Statement** | **Very much disagree** | **Disagree** | **Neutral** | **Agree** | **Very much agree** |
| --- | --- | --- | --- | --- | --- |
| 13. I am willing to work even if I am at risk of infection | ☐ | ☐ | ☐ | ☐ | ☐ |
| 14. I believe the hospital will provide adequate protection | ☐ | ☐ | ☐ | ☐ | ☐ |
| 15. I fear losing my job if I do not report to work | ☐ | ☐ | ☐ | ☐ | ☐ |
| 16. I will not report for duty because I fear getting sick | ☐ | ☐ | ☐ | ☐ | ☐ |
| 17. I will not report because I fear infecting my family | ☐ | ☐ | ☐ | ☐ | ☐ |

**PART IV: Practice Regarding Disaster Preparedness**

1. **In the past one year, have you practiced or drilled what to do during a disaster?**

- ☐ Yes
- ☐ No (Skip next question)

1.1 **How many drills have you participated in?**

1. **Have you participated in disaster management training in your hospital?**

- ☐ Yes
- ☐ No (Skip next question)

2.1 **How many times per year is disaster training provided?**

1. **Have you seen or heard that the disaster plan is periodically updated?**

- ☐ Yes
- ☐ No (Skip next question)

3.1 **How often is the disaster plan updated per year?**

1. **Have you ever faced a real disaster during your emergency department work?**

- ☐ Yes
- ☐ No

1. **Have you ever worked as a member of a disaster management team?**

- ☐ Yes
- ☐ No

1. **Have you taken first aid training (e.g., Basic Life Support) in the past year?**

- ☐ Yes
- ☐ No

1. **Do you believe your practical skills are sufficient to manage disaster events?**

- ☐ Yes
- ☐ No
